# Nutrient Homeostasis of Aegilops Accessions Differing in B Tolerance Level under Boron Toxic Growth Conditions

**DOI:** 10.3390/biology11081094

**Published:** 2022-07-22

**Authors:** Mohd. Kamran Khan, Anamika Pandey, Mehmet Hamurcu, Mateja Germ, Fatma Gokmen Yilmaz, Merve Ozbek, Zuhal Zeynep Avsaroglu, Ali Topal, Sait Gezgin

**Affiliations:** 1 Department of Soil Science and Plant Nutrition, Faculty of Agriculture, Selcuk University, Konya 42079, Turkey; mohdkamran.biotech@gmail.com (M.K.K.); mhamurcu@selcuk.edu.tr (M.H.); fgokmen@selcuk.edu.tr (F.G.Y.); merve12669@gmail.com (M.O.); avszuhal@gmail.com (Z.Z.A.); sgezgin@selcuk.edu.tr (S.G.); 2 Biotechnical Faculty, University of Ljubljana, SI-1000 Ljubljana, Slovenia; mateja.germ@bf.uni-lj.si; 3 Department of Field Crops, Faculty of Agriculture, Selcuk University, Konya 42079, Turkey; atopal@selcuk.edu.tr

**Keywords:** Aegilops, boron toxicity, nutrient uptake, genetic resources, genetic variation, stress tolerance, wheat, wild species

## Abstract

**Simple Summary:**

Boron toxicity stress is known to obstruct the uptake of nutrients in plants inhibiting their proper growth. Thus, the root-shoot nutrient homeostasis of plants under B toxicity stress is an important criterion that should be focused on understanding nutrients’ role in providing tolerance to tolerant genotypes under excess B. Thus, in this study, the effect of toxic and highly toxic B on the nutrient uptake of Aegilops genotypes differing in B tolerance was studied for the first time. The results suggested that the readjustment of nutrient element levels in root-shoot tissues under high B stress are attributable to improved B toxicity tolerance. The information could be used to alleviate high B stress symptoms in modern wheat cultivars via breeding programs.

**Abstract:**

Boron (B) is a crucial microelement for several biological processes in plants; however, it becomes hazardous when present in excess in the soil. B toxicity adversely affects the wheat yield all around the world, particularly in the arid and semiarid regions. Aegilops, the nearest wild wheat relatives, could be an efficient source to develop B toxicity tolerance in modern cultivars. However, to potentially utilize these species, it is necessary to understand the underlying mechanisms that are involved in providing them tolerance. Other than hampering cellular and physiological activities, high B inhibits the uptake of nutrients in wheat plants that lead to nutrients deficiency causing a hindered growth. Thus, it is crucial to determine the effect of B toxicity on nutrient uptake and finally, to understand the role of nutrient homeostasis in developing the adaptive mechanism in tolerant species. Unfortunately, none of the studies to date has explored the effect of high B supply on the nutrient uptake in B toxicity tolerant wild wheat species. In this study, we explored the effect of 1 mM B (toxic B), and 10 mM B (very toxic B) B on the nutrient uptake in 19 Aegilops genotypes differing in B tolerance in contrast to Bolal 2973, the familiar B tolerant genotype. The obtained outcomes suggested a significant association between the B toxicity tolerance and the level of nutrient uptake in different genotypes. The B toxicity tolerant genotypes, Ab2 (TGB 026219, *A. biuncialis* genotype) and Ac4 (TGB 000107, *A. columnaris* genotype) were clustered together in the nutrient homeostasis-based heat map. Though B toxicity mostly had an inhibitory effect on the uptake of nutrients in root-shoot tissues, the tolerant genotypes revealed an increase in nutrient uptake under B toxicity in contrast with Control. The study directs towards future research where the role of external supply of few nutrients in enhancing the B toxicity tolerance of susceptible genotypes can be studied. Moreover, the genotype-dependent variation in the nutrient profile of the studied Aegilops genotypes under high B suggested that increasing number of Aegilops germplasm should be screened for B toxicity tolerance for their successful inclusion in the pre-breeding programs focusing on this issue.

## 1. Introduction

Boron (B), a nonmetallic micronutrient, is involved in different growth and biological processes of vascular plants including structural formation of cell wall, normal functioning and integrity of plasma membrane, lignin biosynthesis, xylem differentiation, sugar transport, and seed development [1]. Due to its participation in different processes, both its deficiency and toxicity are detrimental for plant growth. Under normal B condition in soil, it is usually uptaken by plants as boric acid through passive diffusion, however, in B toxic environment, borate exporters and boric acid channels facilitate B movement in plants [2]. Although boron is necessary for plants, its optimal range is constrained. For semi B-tolerant plants such as wheat, the maximum permitted B values in irrigation water range from 1–2 mg L^−1^. Moreover, the acceptable B levels vary depending on the crop species and genotypes. For instance, B concentrations in dicots range from 20 and 70 mg/kg, while those in the majority of monocots are between 1 to 6 mg/kg. The environmental variables such as light intensity, humidity and rainfall, and numerous other aspects such as soil organic material, lime content, pH, the makeup of clay minerals, soil texture, and interactions with other elements, all have an impact on the availability of boron in soil, making it toxic for plants. Due to the evaporation of ground water and deposition of excessive B in topsoil, B toxicity in soil takes place. Boron toxicity usually occurs in dry areas when plants are cultivated on volcanic or marine-derived alkaline soils. B toxicity in soil also occurs due to B mining and processing, irrigation water, wastewater, fertilizer and herbicide application. Ultimately, plants absorb this high B from the soil and this excess B causes enhanced accumulation of B in symplast and apoplast of plant cells disturbing the significant activities such as carbohydrate metabolism, photosynthesis, stomatal conductance, cell division, antioxidant enzyme activities, and water uptake [3,4,5]. Hampering of these activities lead to the development of symptoms such as chlorosis and necrosis of leaf tips, delayed foliation, and decline in dry matter production [6].

B toxicity negatively affects the agricultural yield worldwide, especially in the arid and semiarid regions [7]. Wheat, a significant food crop, is found to be hugely influenced by excess B in different countries including Turkey, India, Pakistan, Russia, US, Chile, Peru, Italy, Morocco, Israel, Australia, and Serbia [8,9,10,11]. An expanded wheat germplasm is a prerequisite to develop the genotypes that may adapt to B toxic environment [12]. Moreover, to efficiently utilize potential sources from different wheat gene pools that are tolerant to B toxicity, it is necessary to understand the underlying mechanisms that can be involved in providing them tolerance. As B interacts with other mineral nutrients during different processes, a comprehensive understanding of ionic homeostasis is crucial for the upgrading of tolerance to B toxicity in wheat crops.

Several studies reported that excess B disturbs the nutrient uptake and leads to an ion imbalance in plants. High B in growth medium affects the cation-anion values in plants. B toxicity is found to be related to a high ratio of Ca + Mg/K and Ca + K/Mg in plants [13]. On one hand, high B has been reported to increase Mn, Fe, Cu, and Zn in bush bean tissues [14], while on other hand, high B had been reported to decrease Mn, Fe, Cu, Zn, Ca, N, P, and K of maize plants [15]. Similarly, an antagonism was reported between B and leaf P and N in tomato [16], between B and Ca and Mg in wheat and gram crops [17], and between B and Ca and Mg in lentil plants [18]. In wheat, increasing B was reported to decrease the Mn, Fe, Cu, K, and Ca concentrations and increase the Zn, Mg, and P concentrations via impairment in the nutrient absorption process [19]. An increase in the concentration of Zn and Cu and a decrease in the concentration of Fe, M, and Mo, was reported under high B in radish plants [13]. An increase in N, P, and K content in tomato plants was determined under B toxic conditions. B is considered as a component of interactions between two or more than two elements such as P-Mg, Ca-Mg, Cu-Mn, and Ca-P [20,21,22]. It can be concluded from the previously published studies that B plays a significant role in nutrient interactions within plants. Nevertheless, due to a complex nature of nutrient interactions, the effect of high B on nutrient uptake is largely variable and need to be individually explored in different species and even genotypes. As evident from literature, limited studies have been conducted to determine this effect in wheat and unfortunately, no such study is available on Aegilops.

Aegilops genus comprises of 22 different species that are present in the secondary and tertiary gene pool of wheat. Despite the recombination barriers, several Aegilops species have been successfully used to develop tolerance against different stress conditions in the form of introgression/translocation lines. However, efforts to explore or utilize Aegilops species for improving B toxicity tolerance in modern wheat cultivars have been limited [23]. Thus, in this direction, in one of our previous studies, 19 Aegilops accessions from six different species along with B toxicity tolerant cultivar, Bolal, were screened for variation in tolerance towards high B. Other than the considerable dissimilarity among the studied accessions in terms of proline accumulation, lipid peroxidation, and growth parameters, two of the genotypes, Ab2 (TGB 026219, *A. biuncialis*) and Ac4 (TGB 000107, *A. columnaris*), were found to be tolerant to highly toxic (10 mM) B.

In the present study, we explore the effect of high B stress on the accumulation of different macro and micronutrients in the roots and shoot of the studied accessions. The aim is to understand whether there is any association among the B toxicity tolerance and the level of uptake of different nutrients in different genotypes. This information could provide an insight into the role of nutrients in providing tolerance to Aegilops species under excess B and could be used to alleviate high B stress symptoms in modern wheat cultivars via breeding programs.

## 2. Materials and Methods

Seeds of 19 Aegilops genotypes that were obtained from AARI National Gene Bank, İzmir, and Turkish Seed Gene Bank (TSGB), Ankara, Turkey as well as well-known B toxicity tolerant hexaploid wheat genotype, Bolal 2973 [24,25], were used to conduct this study (Table 1). The material comprising of six diverse species along with the check cultivar was previously screened for B toxicity tolerance levels in terms of growth parameters, lipid peroxidation and proline analysis [26].

### 2.1. B Stress Induction and Harvest at Tillering Stage

For the trial, initially, seeds were disinfected employing sodium hypochlorite, and 75% ethanol, and further rinsed with ultrapure water before keeping it in the dark for 3 days at 22 °C for germination. Three-days-old seedlings were transplanted in surface sterilized hydroponic pots containing 1/5th Hoagland’s solution employing a random design, and the temperature, light intensity, light/dark photoperiod, and humidity of the chamber was calibrated to 22 ± 10 °C, 16,000 Lx/day, 16/8 h, and 45–55%. Following the growth of 3 days, plants in hydroponic pots were provided with Control, toxic B, and highly toxic B treatment. The trial comprised of three replicates for each of the three different treatments (Control (1/5th Hoagland solution with 3.1 μM B), 1 mM B (toxic B, 10 ppm), and 10 mM B (very toxic B, 100 ppm)). Five individual plants were present in each replicate making 15 plants per genotype for every B treatment and these 15 plants were pooled at the later stage before taking the ICP-AES measurements. The B treatment was sustained for 7 days, and following every 3 days, the nutrient solutions were swapped with a fresh batch of solutions at the intended B levels. Following 7 days of B treatment, plants were harvested at the tillering stage (Feekes scale 4–5) and root-shoot samples were collected in triplicates for further measurements and analysis.

### 2.2. ICP-AES Analysis

After harvest, growth parameters of the harvested root-shoot samples were estimated as mentioned by Khan et al. [26]. For ICP-AES, from the same trial, root-shoot samples were cleaned with 0.1 N HCl and ultrapure water, and dried at 70 °C for 72 h followed by the pooling of the replicates and measurement of RDWs (root dry weight) and SDWs (shoot dry weights). Dried root-shoot tissues were powdered and 5 mL and 2 mL of 65% HNO_3_ and 35% H_2_O_2_, respectively, were used to liquefy 0.15–0.20 g of the powder. Following the digestion of the mixture in a sealed microwave accelerating reaction machine (Cem Marsxpress, Matthews, NC, USA), the nutrient amount in the stock solution was estimated employing ICP-AES (Varian, Vista, Palo Alto, CA, USA) [27]. The licensed values of elements in the reference leaf material supplied by the NIST, Gaithersburg, MD, USA (National Institute of Standards and Technology) were used to inspect the obtained elemental concentrations.

### 2.3. Data Analysis

The obtained ICP-AES data for different nutrients and the tissues’ dry weight was used to estimate the uptake of nutrients in root and shoot tissues. The nutrient uptake was calculated as below

Root/Shoot Nutrient uptake (mg plant^−1^) = Root/Shoot Nutrient concentration (mg g^−1^) × Root/Shoot dry weight (g plant^−1^)

Statistical analysis of the obtained nutrients’ uptake data was conducted to determine the significance of the effect of different B toxic treatments on the nutrient uptake of different genotypes. The percentage changes in uptake of all the elements in toxic B and highly toxic B treatment in contrast to Control conditions was determined employing MS Excel 2010. GraphPad prism 9.0 program was employed to perform two-way ANOVA on the acquired data where the studied nutrient was the response and genotypes and treatments were the two factors. The values with *p* < 0.001 were accepted to be considerably significant to understand the effect of genotypes (G) and treatments (T) on the variance in elemental uptake. Dunnett’s multiple comparisons test was employed to estimate the average difference between the 1 mM and 10 mM B supply in comparison with the Control treatment, and the outcomes with *p* < 0.001 were accepted to be significantly different. The variation in the uptake of different elements in Aegilops genotypes due to B toxicity stress has been portrayed via Bar diagrams. The correlation among the relative changes in the elements and growth parameters in B toxicity was determined using Pearson correlation- based correlation analysis of Minitab (version 16, State College, PA, USA) software and the correlations were accepted to be significant when *p* < 0.05. Using the average linkage approach, Euclidean distance matrix of the percentage changes in the nutrient values and growth parameters was used to draw a heat map. The heat map presented the grouping of the studied genotypes according to their reaction towards high B and the clustering of nutrients and growth parameters based on their relationship with one another.

## 3. Results

This experiment aims to observe the effect of two different B toxicity treatments on the accumulation of different elements in 19 studied Aegilops accessions together with a B toxicity tolerant wheat genotype, Bolal 2973. These accessions belong to six different Aegilops species counting *A. biuncialis*, *A. columnaris*, *A. ligustica*, *A. speltoides*, *A. triuncialis*, and *A. umbellulata*. Moreover, it is aimed to comprehend the importance of nutrient uptake in imparting high B tolerance to these Aegilops accessions by determining the correlation between the different studied nutrients and the previously published growth parameters based on data from the same hydroponic trial [26].

### 3.1. ANOVA for All the Studied Root-Shoot Nutrients

Based on ANOVA, a substantial genotypic effect was observed on the accumulation of different nutrients except the root sodium (RNa) and root-shoot boron (RB and SB) (Table 2). These significant disparities in nutrient uptake highlight a variable nutrient uptake/accumulation mechanism in the studied accessions. Similarly, B treatment was found to have significant contribution in variability of root phosphorus (RP), shoot sodium (SNa), root magnesium (RMg), shoot manganese (SMn), shoot copper (SCu), root boron (RB), and shoot boron (SB). Dunnett’s multiple comparisons test showed that none of the studied nutrients showed significant mean difference among Control and 10 ppm B supply. Though the mean difference among Control and 100 ppm treatment was significant for different nutrients including RP, SNa, RMg, SMn, RCu, SCu, RB, and SB (Table 2). Thus, the nutrients with significant impact of 10 mM B toxicity treatment on variability are mainly focused on while discussing the results.

### 3.2. Effect of Highly Toxic B on the Root Phosphorus Uptake

The studied Aegilops accessions revealed extensive diversity in RP uptake in Control treatment ranging from 0.0002 to 0.1682 mg per plant, and in 10 mM B treatment ranging from 0.015 to 0.155 mg per plant (Table S1). The check genotype Bolal 2973 revealed a 76% decrement with 0.132 mg per plant RP under Control treatment, and 0.075 mg per plant RP in 10 mM treatment. While Ab2 (100%) and Ab3 (48%) revealed the highest increase in RP uptake, As2 (−457%), and Ac2 (−313%) revealed the highest decline in RP uptake in contrast to the check genotype (Table 3). Referring to the ANOVA results, both treatment (T) and genotypes (G) behave as a significant cause of variability in RP uptake (Table 2).

### 3.3. Effect of Highly Toxic B on the Shoot Sodium Uptake

In Control treatment, the average SNa uptake of Aegilops accessions varied from 0.007 to 0.036 mg per plant, while it ranged from 0.015 to 0.039 mg per plant in highly toxic B treatment (Table S1). Amid Aegilops genotypes, only As2 revealed a decrement (47%) in SNa uptake in 10 mM B treatment in comparison to the Control treatment, while all the other genotypes showed an increment with a maximum increase in Au2 (76%). The check genotype Bolal 2973 revealed a gain of 51% in SNa uptake (Table 3). ANOVA results revealed a significant (*p* < 0.0005) and highly significant (*p* < 0.0001) difference in genotypes and B toxicity treatment (Table 2).

### 3.4. Effect of Highly Toxic B on the Root Magnesium Uptake

The RMg uptake of Aegilops genotypes fluctuated between 0.006 to 0.063 mg per plant in controlled environment, while it varied from 0.005 to 0.036 mg per plant in 10 mM B supply (Table S2). High B toxicity established due to 10 mM B caused a decrease in RMg uptake in the range of 4% to 404% in Aegilops accessions except Ab3, At1, and At2 genotypes that showed an increment of 32%, 42%, and 17%, respectively, in comparison to Control treatment. Bolal 2973 showed a reduction of 91% in 10 mM B supply in comparison to Control (Table 3). Referring to the ANOVA results, both treatment (T) and genotypes (G) significantly (*p* < 0.0005) contributed to the variability among the accessions (Table 2).

### 3.5. Effect of Highly Toxic B on the Shoot Manganese Uptake

A large variability in the SMn uptake of Aegilops genotypes was noticed in both Control (0.99–5.21 µg per plant) and highly toxic B supply (0.85–4.10 µg per plant) (Table S2). SMn uptake in Bolal 2973 was 4.49 and 4.71 µg per plant in Control and highly toxic B supply, respectively, showing an increment of 5% (Table 3). All the Aegilops accessions showed a decrement in SMn uptake in the range of −1% to −350% in a highly toxic B supply in comparison to Control except AC4 which showed 40% increment (Table 3). In case of SMn uptake, treatment and genotypes served as a significant source of variability. However, the genotypes (*p* < 0.0001) revealed a much larger impact on the variability as compared to treatments (*p* < 0.005) (Table 2).

### 3.6. Effect of Highly Toxic B on the Root Copper Uptake

The RCu uptake of Aegilops accessions varied from 0.56 to 7.82 µg per plant and 0.63 to 5.34 µg per plant in Control and highly toxic B supply, respectively (Table S3). While the check cultivar Bolal 2973 revealed a decrement of 39% in RCu uptake, Aegilops accessions showed a wide range with Ac1 and Ac2 showing a decrement of 173% and 492%, respectively, and Al1 and At1 showing an increment of 44% and 38%, respectively (Table 3). In RCu uptake, highly significant (*p* < 0.0001) differences were observed in the genotypes which contributed to 82.3% variation. However, non-significant differences were observed in the treatment (Table 2).

### 3.7. Effect of Highly Toxic B on the Shoot Copper Uptake

The SCu uptake of Aegilops genotypes ranged from 0.51 to 3.19 µg per plant in Control treatment, while it varied between 0.57 to 1.64 µg per plant in 10 mM B supply (Table S3). Most of the genotypes revealed a decrement in the SCu uptake in comparison to Control, except As1, At1, At2, Au2, Au3, and Bolal in high N supply. Au2 and At2 showed maximum increment of 37% and 31%, respectively (Table 3). Both treatment (T) and genotypes (G) act as a significant source of variability in SCu uptake of experimental Aegilops accessions (Table 2).

### 3.8. Effect of Highly Toxic B on the Root-Shoot B Uptake

The RB uptake ranged from 0.75 to 17.80 μg per plant in Aegilops accessions in 10 mM B supply (Table S3). Ab3 and Al2 showed maximum and minimum increment in RB uptake in highly toxic B supply in comparison to Control (Table 3). Though treatment contributed significantly to the RB uptake variability, genotypes did not have significant effect on the variation (Table 2).

The SB uptake ranged from 30.52 to 131.24 μg per plant in Aegilops accessions in 10 mM B supply (Table S3). Au2 and Ac5 showed maximum and minimum increment in SB uptake in highly toxic B in comparison to Control (Table 3). Though treatment contributed significantly to the SB uptake variability, genotypes did not have significant effect on the variation (Table 2).

### 3.9. Physiological Differences in the Aegilops Genotypes in Highly Toxic B at the Tillering Stage

At the tillering stage when samples were collected to estimate root-shoot nutrients content and uptake, Aegilops accessions showed significant variation in growth parameters under different B treatments. Though these differences in growth parameters at the tillering stage have been presented in detail in one of our previously published studies (Khan et al. 2021), these have been partially discussed here to understand the association, if any between the growth parameters and the accumulated root-shoot nutrients. The Aegilops accessions, Ab2 and Ac4 that were considered to be tolerant, were found to be least affected by highly toxic B, in terms of root-shoot dry weight (Table S4). Ab2 and Ac4 revealed the maximum increment of 31% and 20% in SDW, respectively, under highly toxic B in comparison to Control (Table 3). Similarly, where all the genotypes showed a decrement in RDW in 10 mM in comparison to Control, these genotypes showed least decrement of 9% in Ab2 and 13% in Ac4 (Table 3).

### 3.10. Correlation among the Root-Shoot Nutrient Uptake of Aegilops Genotypes under Highly Toxic B Supply and Their Association with the Growth Parameters

Pearson’s correlation was used to estimate the correlation among the comparative changes in the root-shoot nutrient uptake in 10 mM B supply in comparison to Control (Table 4). RB showed significant or highly significant positive associations with RP, SP, RNa, and SB. Similarly, SB was determined to be significantly correlated with SCa, SP, SMg, and SMn. In roots, Ca was found to be significantly correlated with P, Na, Mg, Mn, and Cu. In the same way, in shoots, Ca was found to be significantly associated with P, Na, Mg, Mn, and B. RP revealed a strongly significant association with SNa, RNa, RMg, RMn, and RB. However, SP was significantly associated with SNa, RNa, SMg, SMn, SB, and RB. RNa was significantly associated with SMg, RMg, RMn, and RB, while SNa was significantly correlated with RNa, SMg, SMn, and RMn. In roots, Mg had a significant positive correlation with Mn and Cu, while in shoots, Mg showed significant positive association with Mn and B. A significant positive correlation was detected between SMn and SB and between RMn and RCu. Interestingly, SCu was not significantly correlated with examined nutrients and growth parameters at all. While evaluating the association between the growth parameters and different nutrients’ uptake, it was determined that RDW was significantly correlated with SP, RP, and RNa, while SDW was significantly associated with SP, RNa, SMg, SMn, SB, and RB. Moreover, all the identified significant associations in the study were positive (Table 4).

### 3.11. Hierarchical Cluster Analysis-Dependent Heat Map

**Figure 1:**
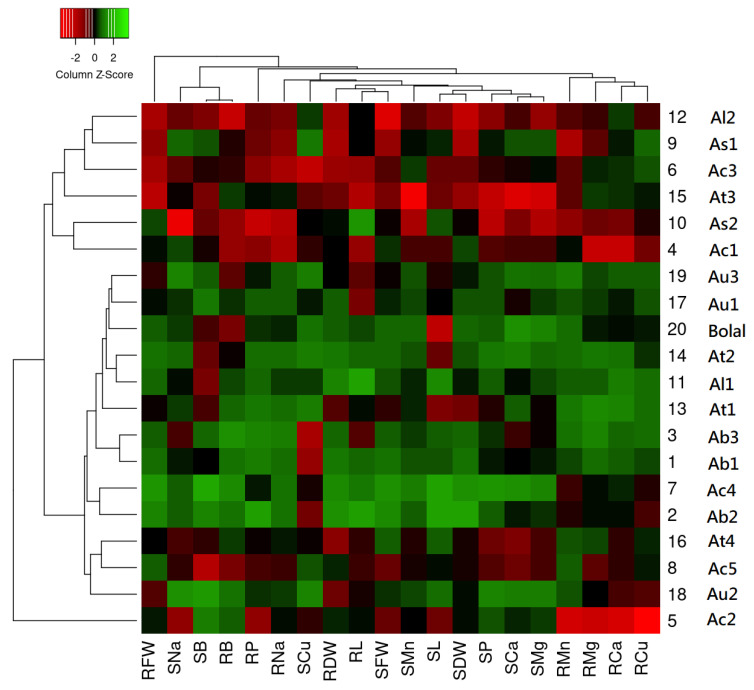
Heat map showing the genetic variation of 19 Aegilops genotypes together with the check genotype, Bolal 2973, based on the nutrient uptake and the previously studied growth parameters under high B (10 mM) treatment. The color indicates the Z score for each accession: dark green shows the increment in the percentage of the nutrient uptake values of the genotypes in highly toxic B in comparison to control, whereas red shows the decrement in the percentage of the nutrient uptake of the genotypes in B toxicity in comparison to control. The accessions were grouped into two different clusters (C1–C2) with C1 divided into two subclusters, C1A and C1B, according to their reaction to highly toxic B in terms of several previously studied traits in Khan et al. (2021) including RL (root length), SL (shoot length), RFW (root fresh weight), SFW (shoot fresh weight), RDW (root dry weight), SDW (shoot dry weight), and the nutrient uptake estimated in this study including RCa (root calcium), SCa (shoot calcium), RP (root phosphorus), SP (shoot phosphorus), RNa (root sodium), SNa (shoot sodium), RMg (root magnesium), SMg (shoot magnesium), RMn (root manganese), SMn (shoot manganese), RCu (root copper), SCu (shoot copper), RB (root boron), and SB (shoot boron) uptake.

The heat map shown in Figure 1 illustrates the effect of highly toxic B treatment on the nutrient profiles of Aegilops genotypes along with the Bolal 2973 as compared to Control treatment. The percentage changes in nutrients and growth parameters in 10 mM B supply in comparison to Control distributed the studied genotypes into two main clusters, Cluster 1 and Cluster 2, with the one out group genotype, Ac2. Cluster 1 was further sub-grouped in Cluster 1A and 1B. Cluster 1A consists of nine Aegilops genotypes and Bolal. The genotypes in Cluster 1A largely revealed the green background showing that most of the presented parameters (both nutrients and growth parameters) showed increments in high B supply in comparison to Control. Cluster 1B consists of 3 genotypes and it largely revealed the mix of green and red background showing that some of the presented parameters showed increments while some of them showed decrements on applying high B. Cluster 2 comprises six accessions that largely revealed red background presenting that most of the presented parameters in this group showed decrements in high B supply in comparison to Control. In addition, the heat map also categorized the presented parameters into three separates clusters. Cluster A consists of root accumulated nutrients including RMn, RMg, RCa, and RCu. Cluster B consists of both root and shoot related parameters. Close association of RB and SB in Cluster C was in accordance with the Pearson correlation analysis.

## 4. Discussion

The process of domestication has had intense deteriorating effects on the genetic variation of the modern wheat gene pool. The immediate wheat relative, Aegilops, which fits in its tertiary gene pool, holds enormous potential for providing unique alleles to enhance this genetic variability in wheat. Regardless of the crossability and incompatibility issues, Aegilops species are being successfully used to develop tolerance against different abiotic and biotic stress conditions along with the other adaptive quality traits [26,28,29,30,31,32,33,34,35,36].

Despite lots of efforts towards employing Aegilops for different abiotic constraints, very less information is available on the B toxicity tolerance of Aegilops species. Although in the past few years, some studies described the disparity in B toxicity tolerance of different Aegilops species and genotypes [23,26], none of these focused on the uptake of nutrients in Aegilops species under B toxicity. Furthermore, the possible role of changes in nutrient uptake in providing B toxicity tolerance to them has not been examined. However, a thorough understanding of nutrients’ homeostasis under B toxicity is crucial for the upgrading of B toxicity tolerance in crops. Thus, we determined the changes in the nutrient uptake of 19 Aegilops accessions and their differing B toxicity tolerance under high B supply as compared to Control treatment. The aim of this study is to understand the underlying mechanism and determine whether the extent of accumulation of different nutrients in the root-shoot tissues under B toxic conditions is associated with the tolerance level of genotypes or not.

### 4.1. Effect of Highly Toxic B on the Root Phosphorus Uptake

**Figure 2:**
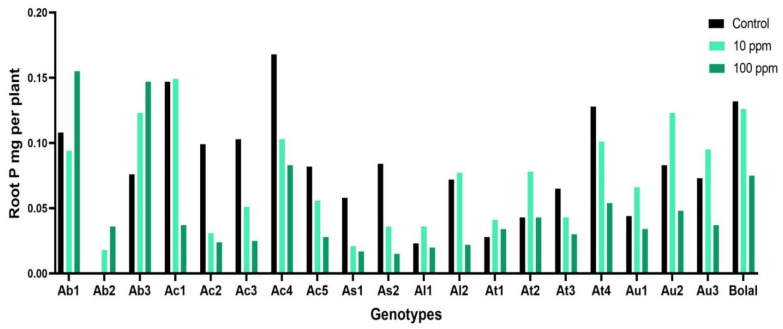
Variation in the RP (root phosphorus) uptake of 19 Aegilops genotypes and Bolal 2973, the B tolerant check genotype in Control (3.1 μM B), 10 ppm B (toxic B), and 100 ppm B (highly toxic B).

Phosphorus, which is found in every living plant cell, is crucial for plant growth. Being involved in cellular formation of ATPs and being an important component of nucleic acids, it is part of key processes in plants such as conversion of starches and sugars, photosynthesis, energy transfer, and protein synthesis. Several studies suggested that the interaction between B and P is greatly important for crop plants [37,38]. In different previous studies, B toxicity has been shown to have reducing effect on P uptake in plant tissues [19,38,39,40]. In accordance with the previous literature, in our study also, most of the Aegilops genotypes showed a decrement in root P uptake (Figure 2). Even the check cultivar, Bolal 2973, showed a decrement of 76%. The decrement can be explained by the fact that both borate and phosphate ions are actively taken up by the plants following the same mechanism, and there is a competition between the two. It means higher concentration of B inhibited the uptake of P ions. This was against the obtained positive correlation between P and B uptake in plant tissues (Table 4).

However, contrary to the above mentioned concept, few accessions in our study showed an increment in root P uptake under B toxicity (Table 3) showing the effect of genotype on the trait. The two Aegilops accessions with maximum increment in RP uptake also showed high B uptake (Table 3). This was in line with the obtained positive correlation between P and B uptake in plant tissues (Table 4). These differences in the P uptake of genotypes direct towards some other underlying mechanisms that need to be thoroughly explored.

### 4.2. Effect of Highly Toxic B on the Shoot Sodium Uptake

Although sodium is not considered as an ‘essential’ element for wheat, it may have different benefits at times due to its chemical and structural similarity with potassium ions in hydrated forms [41]. Thus, Na+ can fulfill many of the biophysical functions of K+ such as maintaining cell turgor [42]. Most of the dry regions of the world suffer from high B and salinity stress simultaneously and thus, the combined B and salt (BorSal) stress condition is now being considered as a novel stress condition [2]. Accordingly, a relevant number of studies that have observed the BorSal effect on Na or B accumulation in tissues are available. Few studies have shown positive effects of BorSal stress on plants while others have shown the negative effects. It means the interaction of B and Na uptake within the plant tissues is not conclusive. Moreover, the effect of boron on sodium uptake/accumulation has been an overlooked topic. However, it would be interesting to explore the interaction of both the elements under B toxicity, as this may help in understanding the contrasting responses of combined BorSal stress and individual stress on plant growth, and accordingly, solutions can be found to reduce the devastating effect on crops. Accumulation of Na in plant tissues is considered as an important attribute for understanding the adaptive mechanism for different stress conditions especially salinity and water stress. However, the influence of highly toxic B on root-shoot Na accumulation in plants, especially wheat, has not been well explored.

**Figure 3:**
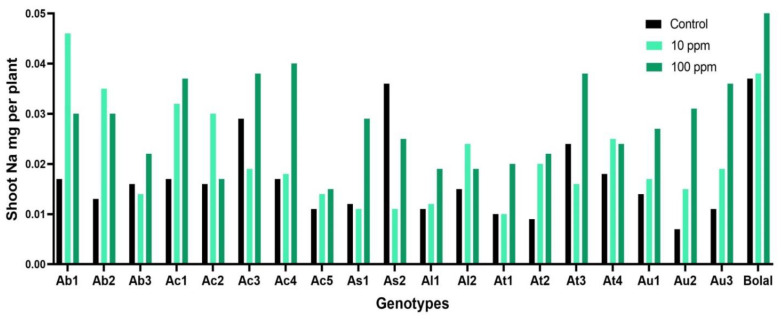
Variation in the SNa (shoot sodium) uptake of 19 Aegilops genotypes and the B tolerant check cultivar, Bolal 2973, in Control (3.1 μM B), toxic B (10 ppm B), and highly toxic B (100 ppm B).

There are very few studies focusing on the effect of B toxicity on the SNa uptake in wheat. In the present study, most of the genotypes revealed an increment in SNa uptake except As2 (Figure 3). Our results were in agreement with Javid et al. [43], where two differently B tolerant *Brassica juncea* genotypes showed a gain in SNa uptake under high B irrespective of their tolerance level towards B toxicity.

These results were contrary to Tepe and Aydemir [44] who found a significant decrement in SNa of both tolerant and susceptible wheat genotype under 10 mM B treatment. Not only in wheat, but also in cucumber [45] and pepper, [46] SNa uptake was found to decrease under high B supply.

### 4.3. Effect of Highly Toxic B on the Root Magnesium Uptake

**Figure 4:**
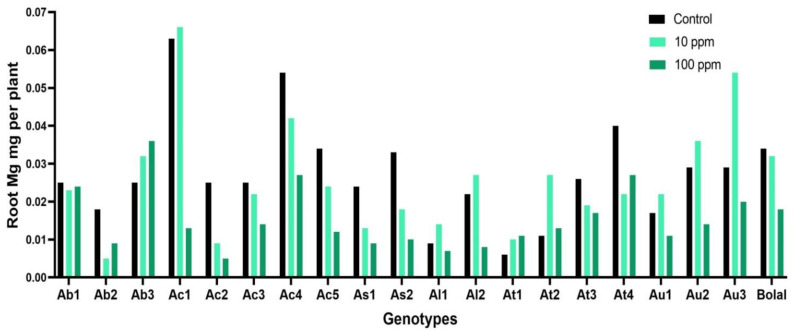
Variation in the RMg (root magnesium) uptake of 19 Aegilops genotypes and Bolal 2973, the B tolerant check genotype, in Control (3.1 μM B), 10 ppm B (toxic B), and 100 ppm B (highly toxic B).

Magnesium is an essential element for plant growth whose role is mainly discussed in terms of photosynthesis in leaf tissues. However, it is equally crucial for root cells for several basic biochemical processes such as carbohydrate metabolism, activation/inactivation of enzymes, synthesis and folding of nucleic acid, and energy generation [47]. In this study, except 3 Aegilops accessions, all other genotypes showed a decrement in the RMg uptake (Figure 4).

Though most of the genotypes showed a decrement, the increment in few genotypes showed that the Mg uptake in root under B toxicity was variable and dependent on the genotype. The decrease in RMg was in agreement with Lovatt and Bates [48] who identified a decrease in the total root Mg of cucumber pepo plants under high B treatment in hydroponic system. Similar to our results of decrement in RMg, Tepe and Aydemir [44] also revealed that both the experimental wheat genotypes showed a decrement in the root Mg under B toxicity irrespective of their tolerance levels toward high B.

### 4.4. Effect of Highly Toxic B on the Shoot Manganese Uptake

**Figure 5:**
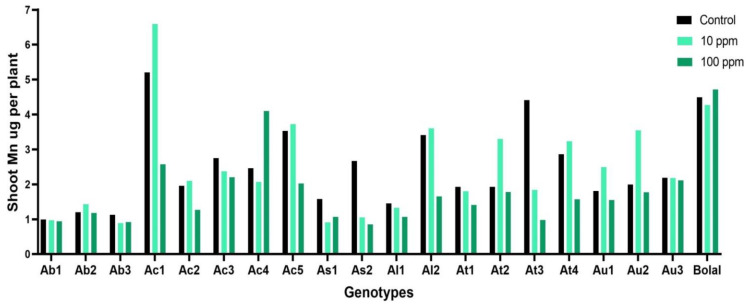
Variation in the SMn (shoot manganese) uptake of 19 Aegilops genotypes and the B tolerant check cultivar, Bolal 2973, in Control (3.1 μM B), toxic B (10 ppm B), and highly toxic B (100 ppm B).

Mn, which is required in a small amount by plants, is one of the essential nutrients for their growth. Besides being an essential factor of the oxygen-evolving complex of the photosystem II (PSII) in plants, it activates over 35 enzymes that are involved in different physiological and metabolic processes, and help plants in developing tolerance against several biotic and abiotic stresses [49,50]. In our study, the check genotype, Bolal 2973, showed an increment in SMn uptake in high B. Similarly, the other two genotypes that were considered as tolerant to high B, Ac4 and Ab2, were shown to have increment and least decrement, respectively, in SMn uptake (Figure 5). Similar to our results of tolerant genotypes, [48] we determined that SMn of *C. pepo* increased after the excess B supply for 72 and 96 h. However, the content was decreased after 120 h supply. Moreover, in a study, Samet et al. 2015 determined that SMn significantly increased under 20 mg per kg B supply in pepper in comparison to Control. Our outcomes were different from the results of Tepe and Aydemir [44], where both tolerant and susceptible genotype showed a decrease in SMn content. The presence of both increase and decrease in the SMn in the genotypes revealed that it was genotype-dependent rather than being subjected to the treatment.

### 4.5. Effect of Highly Toxic B on the Root-Shoot Copper Uptake

**Figure 6:**
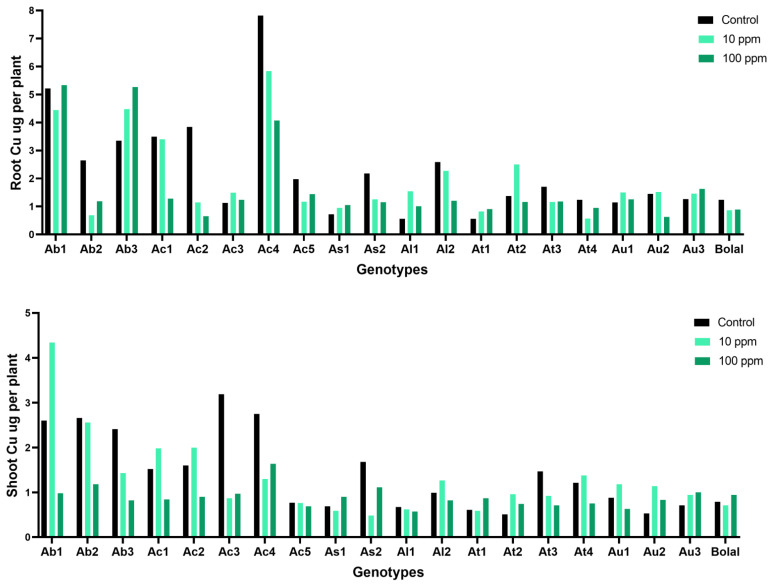
Variation in the RCu (root copper) uptake and SCu (shoot copper) uptake of 19 Aegilops genotypes and Bolal 2973, the B tolerant check genotype in Control (3.1 μM B), 10 ppm B (toxic B), and 100 ppm B (highly toxic B).

Cu, a crucial cofactor of several plant proteins, is required to ensure proper cell functioning. It is mainly involved in regulating the cellular redox state, remodeling of the cell wall, and transport of electrons in chloroplasts and mitochondria [51]. Different abiotic stresses are known to have altered effects on the root-shoot Cu uptake [52]. A highly variable trend was found in the RCu accumulation of the Aegilops accessions where eight genotypes showed an increment while the rest of the genotypes showed a decrement (Figure 6). Similarly, in SCu uptake, five genotypes showed an increase (Figure 6). In both cases, the obtained increase or decrease was not related to the tolerance level of the genotypes. The results were similar to those obtained by Tepe and Aydemir [44] where a decrease in RCu and SCu content was observed in both B tolerant and susceptible wheat genotypes, and the RCu and SCu content was not related to the tolerance level. In *C. pepo*, RCu and SCu content increased as compared to Control under excess B supply for 120 h [48]. Likewise, SCu content was significantly increased in pepper under high B as compared to Control [46].

### 4.6. Effect of Highly Toxic B on the Root-Shoot B Uptake

**Figure 7:**
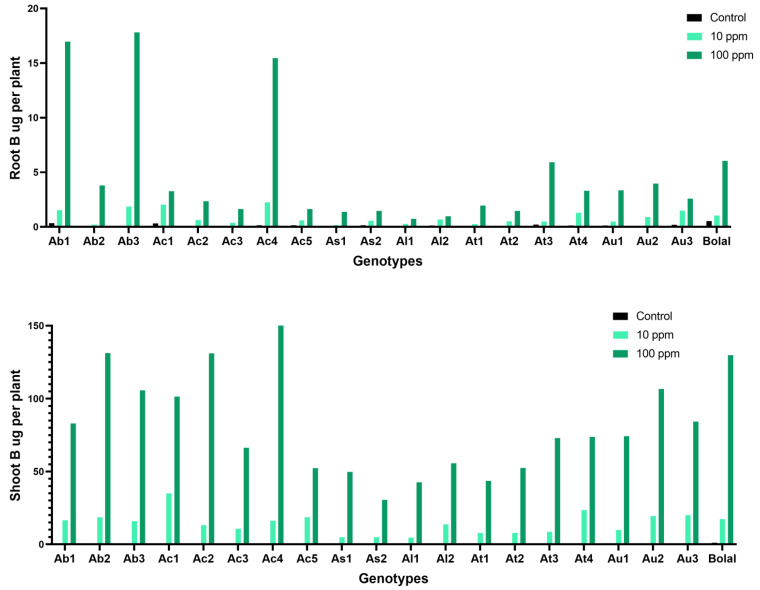
Variation in the RB (root boron) and SB (shoot boron) of 19 Aegilops genotypes and the B tolerant check cultivar, Bolal 2973, in Control (3.1 μM B), toxic B (10 ppm B), and highly toxic B (100 ppm B).

It is generally accepted that the genotypes with lower B accumulation in the tissues can be more tolerant to B toxicity [8,26,53,54,55]. However, several studies revealed that B concentration in the tissues is not correlated with the level of B tolerance in roots and shoots highlighting the underlying mechanisms regulating the process [6,9,56,57,58,59]. The movement of B from the intracellular phase to the apoplast in leaves, which is somewhat less prone to B toxicity, can be one of the mechanisms [26]. In present study, the RB and SB uptake was increased by more than 85% in all the studied Aegilops genotypes together with the check genotype, Bolal 2973. Though one of the previously identified B tolerant genotypes, Ac4, showed highest RB and SB uptake (Figure 7), the increment in RB and SB uptake was not related to the tolerance level of the genotypes. This was in line with the previous studies where higher B accumulation was observed in shoots as compared to roots irrespective of their tolerance levels [44,48].

### 4.7. Association among the Root-Shoot Nutrient Uptake of Aegilops Genotypes under Excess B and Their Association with the Growth Parameters/Heat Map

Here, we discuss the association between the root-shoot nutrient uptake and growth parameters of the Aegilops genotypes under high B. It is crucial to converse that whether the changes in the growth parameters, especially DW, and in turn, the tolerance level of the genotypes, is associated with the comparative changes in the root-shoot nutrients in 10 mM B supply in comparison to Control.

In the present study, strong significant correlations were found among different elements and between the elements and growth parameters. It can be suggested that Aegilops genotypes develop their adaptation to B toxicity stress via nutrients’ homeostasis and the involvement of nutrients in other processes. The tissue dry weight (DW) is considered as an important criterion for determining the wheat genotypes’ tolerance level [26,55,58,60]. In one of the previously published studies, Ab1, Ab2, and Ac4 revealed an increase in SDW, while Ab2 along with Ac4 revealed least decrement in RDW under excess B in comparison to the B tolerant check genotype, Bolal.

In roots, the DW was significantly correlated with SP, RP, and RNa only; while in shoots, the DW was significantly associated with SP, RNa, SMg, SMn, SB, and RB. It means that the SDW was positively associated with three additional elements, Mg, Mn, and B. Mg, being the central core of the chlorophyll molecule, helped in the activation of enzymes and in contributing to protein synthesis required for proper plant growth, while Mn actively participates in processes such as nitrogen assimilation, respiration, cell elongation, pollen tube development, and photosynthesis. B plays an important role in the formation of cell walls, maintaining the integrity of biological membranes, pollination, and transfer of energy into the growing parts of the plants. As these elements are greatly involved in the important functioning of plants, the association of SDW with Mg, Mn, and B can be one of the reasons of less destructive effect of high B on SDWs (2%) than it has on RDWs (20%) [61].

Moreover, the strong significant correlation between the root-shoot dry weight and different nutrients (SP, RP, RNa, SMg, SMn, SB, RB) directs that these elements seem to be involved in providing tolerance to the tolerant genotypes. Both Ab2 and Ac4 have maximum RDW and SDW along with either an increment in these correlated nutrients or a lesser decrement in them under high B treatment as compared to other cultivars. A comparatively less diminishing effect of high B on these nutrients in tolerant genotypes showed their involvement in adaptive mechanism. The B tolerant genotypes, Ab2 and Ac4, showed a moderately high B uptake in root and shoot tissues. The B tolerant genotype, Ac4, showed maximum increment in SCa, SP, RNa, SMg, and SMn. Though the tolerant Ab2 genotype showed maximum increment in RP only, other elements were also less negatively influenced by high B as compared to other genotypes.

A larger detrimental effect of high B on the nutrient uptake of susceptible genotypes also points to the additional supply of these nutrients as supplements which can help in reducing the B toxicity symptoms or may be developing B toxicity tolerance in the susceptible genotypes. Previous reports have also highlighted the alleviation of B toxicity symptoms on external supply of Ca [62], and P [38,63]. The effect of sodium supply on B toxicity symptoms has been widely studied but could not reach a conclusive outcome [2,64,65,66,67,68]. However, the effect of external application of Mg and Mn on inhibiting the negative effects of B toxicity have not been thoroughly explored and should be considered for further research [69]. Not only our knowledge about the effects and mechanisms of different alleviation approaches via nutrient supplementation is limited, but also the supplemental effects of nutrients which should be observed individually and in combination with each other.

### 4.8. Heat Map

A huge variability in B toxicity tolerance levels of Aegilops genotypes in respect of growth parameters, lipid peroxidation, and proline analysis was determined in one of our previous studies [26]. The objective of the present study was to understand whether the nutrient uptake profile of the genotypes under high B stress has a role in developing this variability or providing B toxicity tolerance to the Aegilops genotypes. It is reported that B toxicity stress has a suppressive effect on elemental uptake and accumulation in wheat cultivars. However, Aegilops species have not been well explored [70].

The cluster analysis-based heat map in this study showed there was a large variation in the uptake of nutrient elements in Aegilops tissues at the tillering stage when plants were exposed to B toxicity stress. Although B toxicity stress had a decreasing effect on the uptake of most of the nutrients, few genotypes showed a significant increment in different nutrient uptake in both root and shoot tissues. Cluster 1A comprising nine accessions showed increment in most of the traits, and mostly contain Aegilops accessions with greater B toxicity tolerance including Ab2, Ac4, and the check cultivar, Bolal 2973. Cluster 1B, showing both increment and decrement in different nutrients, is comprised of three moderately B tolerant genotypes (Ac5, At4, and Au2). In Cluster 2, there were six accessions, and most of them showed decrements in nutrient uptake and growth parameters in 10 mM B supply in comparison to Control.

Interestingly, Ab2 and Ac4, that were considered as the tolerant genotypes in our previous study, were closely clustered together in the Cluster 1A. Similarly, four accessions (Al2, As1, Ac3, and At3) that were considered as the susceptible genotypes in our previous study were closely grouped together in the Cluster 2. Even though the heat map obtained in this study was based on the elemental profile and growth parameters of the root-shoot tissues under high B, the clustering of the Aegilops genotypes was much similar to the heat map obtained in our previous study [26] that was based on the growth parameters, lipid peroxidation, and proline analysis. This shows that nutrient uptake under B toxicity stress also influence the overall genetic and functional profile of the genotypes.

It should also be noticed that the grouping based on nutrient uptake and growth parameters in the main or sub-clusters was not according to the ploidy level or species. All the clusters contain genotypes from different ploidy levels and species. Thus, based on the outcomes, it can be concluded that the nutrient uptake in Aegilops genotypes under highly toxic B conditions was genotype-dependent. Consequently, it is necessary to screen more and more Aegilops germplasm for B toxicity tolerance, so that the tolerant accessions can be identified and included in the pre-breeding program for the development of B toxicity tolerance in wheat.

## 5. Conclusions

In summary, the influence of high B stress on nutrient uptake in the root-shoot tissues of 19 Aegilops genotypes differing in B toxicity tolerance was studied to determine the role of nutrient homeostasis in developing the adaptive mechanism in Aegilops species towards B toxicity. Our results indicated that (i) nutrient homeostasis under high B was largely responsible for the high tolerance level of the B toxicity tolerant genotypes, Ab2 (TGB 026219, A. biuncialis genotype), and Ac4 (TGB 000107, A. columnaris genotype). Though B toxicity mostly had a suppressive effect on the nutrient uptake, the tolerant genotypes revealed an increase in nutrient uptake in high B in comparison to Control; (ii) The decrease in the nutrient uptake of susceptible genotypes under high B directs that the external supply of these nutrients can help in developing the B toxicity tolerance in the susceptible genotypes. However, further research is required to confirm this hypothesis; (iii) Large variation in the nutrient profile of the studied Aegilops genotypes under high B, among species and within species, and its association with the B toxicity tolerance suggest that the response was genotype-dependent.

Thus, an increasing number of Aegilops germplasm should be screened for B toxicity tolerance for their successful inclusion in the pre-breeding programs focusing on the issue. As a conclusion, the nutrient analysis of the Aegilops genotypes differing in B toxicity tolerance suggested that readjustment of nutrient element levels in root-shoot tissues under high B stress are attributable to improved B toxicity tolerance. Moreover, it is concluded that root-shoot nutrient profile under B toxicity stress is an important criteria that cannot be ignored while understanding the underlying adaptive mechanism of the Aegilops genotypes.

## Figures and Tables

**Table 1:** Information of the 19 Aegilops genotypes and check hexaploid wheat cultivar, Bolal 2973 used in the study.

GenBank Code	Site of Origin	Abbreviation Code	Ploidy	Genome	Taxon
TGB 026218	Adıyaman, Turkey	Ab1	4x	UUMM	*Aegilops biuncialis*
TGB 026219	Şanlıurfa, Turkey	Ab2	4x	UUMM	*Aegilops biuncialis*
TGB 037313	Gaziantep, Turkey	Ab3	4x	UUMM	*Aegilops biuncialis*
TGB 037373	Gaziantep, Turkey	Ac1	4x	UUMM	*Aegilops columnaris*
TGB 038488	Ankara, Turkey	Ac2	4x	UUMM	*Aegilops columnaris*
TGB 037489	Şanlıurfa, Turkey	Ac3	4x	UUMM	*Aegilops columnaris*
TGB 000107	Adıyaman, Turkey	Ac4	4x	UUMM	*Aegilops columnaris*
TR 57295	Van, Turkey	Ac5	4x	UUMM	*Aegilops columnaris*
TGB 037791	Şanlıurfa, Turkey	As1	2x	SS	*Aegilops speltoides*
TR 62174	Gaziantep, Turkey	As2	2x	SS	*Aegilops speltoides*
TGB 000803	Mersin, Turkey	Al1	2x	SS	*Aegilops ligustica*
TR 39488	Şanlıurfa, Turkey	Al2	2x	SS	*Aegilops ligustica*
TGB 037311	Şanlıurfa, Turkey	At1	4x	CCUU	*Aegilops triuncialis*
TGB 037355	Adıyaman, Turkey	At2	4x	CCUU	*Aegilops triuncialis*
TGB 037376	Gaziantep, Turkey	At3	4x	CCUU	*Aegilops triuncialis*
TR 72224	Adıyaman, Turkey	At4	4x	CCUU	*Aegilops triuncialis*
TGB 037353	Erzincan, Turkey	Au1	2x	UU	*Aegilops umbellulata*
TGB 037356	Şanlıurfa, Turkey	Au2	2x	UU	*Aegilops umbellulata*
TR 72200	Şanlıurfa, Turkey	Au3	2x	UU	*Aegilops umbellulata*
Bolal 2973	Turkey	Bolal	6x	AABBDD	*Triticum aestivum*

**Table 2:** The two-way analysis of variance (ANOVA) to understand the effect of genotypes (G) and treatments (T) on the variance in elemental uptake. Significant differences are presented as **** for *p* < 0.0001; *** for *p* < 0.0005; ** for *p* < 0.005; * for *p* < 0.05; ns, non-significant.

Studied Traits	Code	% of Total Variation		*p*-Value		Control vs. 10 ppm		Control vs. 100 ppm	
		Genotypes	Treatment	Genotypes	Treatment	Mean Difference	Adjusted *p*-Value	Mean Difference	Adjusted *p*-Value
Root Calcium	RCa	60.4	3.7	***	ns	0.010	ns	0.017	ns
Shoot Calcium	SCa	53.5	1.8	*	ns	0.002	ns	0.017	ns
Root Phosphorus	RP	59.6	11.1	***	**	0.007	ns	0.033	**
Shoot Phosphorus	SP	72.8	2.4	****	ns	−0.007	ns	0.048	ns
Root Sodium	RNa	46.6	2.3	ns	ns	0.005	ns	0.003	ns
Shoot Sodium	SNa	48.2	20.2	**	****	−0.004	ns	−0.013	****
Root Magnesium	RMg	54.6	15.9	***	***	0.002	ns	0.012	***
Shoot Magnesium	SMg	73.5	1.7	****	ns	0.000	ns	0.009	ns
Root Manganese	RMn	72.5	2.3	****	ns	0.026	ns	0.240	ns
Shoot Manganese	SMn	67.7	7.3	****	**	0.014	ns	0.712	*
Root Copper	RCu	82.3	2.5	****	ns	0.321	ns	0.600	*
Shoot Copper	SCu	51.6	8.9	**	*	0.111	ns	0.518	*
Root Boron	RB	27.1	31.1	ns	****	−0.728	ns	−4.649	****
Shoot Boron	SB	15.0	58.7	ns	****	−13.790	ns	−88.590	****

**Table 3:** The percentage changes in the uptake of all the studied elements in 19 Aegilops genotypes and the B tolerant check cultivar, Bolal 2973, in highly toxic B treatment in contrast to Control conditions.

Code	SDW	RDW	SCa	RCa	SP	RP	SNa	RNa	SMg	RMg	SMn	RMn	SCu	RCu	SB	RB
Ab1	6	−28	−20	20	−28	31	45	50	−20	−4	−5	−21	−167	2	99.26	98.08
Ab2	31	−9	−13	−37	4	100	58	58	−12	−95	−1	−104	−124	−123	99.61	98.18
Ab3	0	−47	−39	26	−17	48	27	78	−32	32	−24	33	−192	36	99.50	99.36
Ac1	−7	−92	−42	−251	−78	−300	53	−212	−49	−395	−103	−65	−80	−173	99.20	90.11
Ac2	−20	−76	−11	−287	−2	−313	4	−27	−7	−404	−55	−393	−78	−492	99.58	97.29
Ac3	−48	−192	−29	−10	−62	−303	22	−204	−23	−76	−25	−156	−229	10	99.17	93.70
Ac4	20	−13	38	−17	50	−102	58	58	26	−100	40	−131	−68	−92	99.81	99.08
Ac5	−30	−79	−57	−74	−80	−195	30	−71	−49	−188	−75	7	−11	−37	98.64	91.35
As1	−82	−206	5	−29	−30	−242	60	−158	0	−183	−48	−300	23	32	99.43	94.02
As2	−27	−90	−69	−131	−170	−457	−47	−236	−117	−210	−213	−241	−52	−89	99.03	90.12
Al1	−19	−20	−19	56	1	−17	41	13	−5	−25	−35	9	−19	44	98.95	96.66
Al2	−98	−193	−45	−5	−120	−227	20	−130	−91	−156	−107	−152	−21	−116	98.91	86.93
At1	−54	−131	6	60	−55	16	50	48	−33	42	−37	41	30	38	99.09	97.48
At2	−4	−28	26	37	24	1	61	53	8	17	−9	29	31	−18	99.02	94.73
At3	−71	−149	−145	−11	−181	−115	37	−19	−146	−54	−350	−165	−108	−44	98.94	96.28
At4	−28	−175	−71	−72	−94	−137	26	−19	−50	−44	−81	−11	−62	−31	99.15	96.20
Au1	−6	−49	−30	−27	−3	−27	48	31	−10	−54	−17	−9	−40	9	99.56	96.07
Au2	−20	−148	24	−87	35	−71	76	8	20	−109	−12	−10	37	−131	99.72	97.99
Au3	−19	−96	18	19	−5	−100	71	37	11	−45	−4	52	29	23	99.46	92.57
Bolal	0	−53	38	−36	−1	−76	51	−9	24	−91	5	25	16	−39	99.09	91.34

**Table 4:** The association between the relative changes in the elements and root-shoot dry weight in high B treatment (10 mM) as compared to control using Pearson correlation-based correlation analysis. The associations with *p* < 0.05 and *p* < 0.01 were accepted to be significant (*) and highly significant (**), respectively.

T Parameter	SDW	RDW	SCa	RCa	SP	RP	SNa	RNa	SMg	RMg	SMn	RMn	SCu	RCu	SB
RDW	0.85 **														
*p*-value	0.000														
SCa	0.39	0.31													
*p*-value	0.089	0.187													
RCa	−0.09	0.07	0.15												
*p*-value	0.696	0.764	0.519												
SP	0.60 **	0.51 *	0.85 **	0.15											
*p*-value	0.005	0.021	0.000	0.521											
RP	0.44	0.49 *	0.29	0.64 **	0.53 *										
*p*-value	0.053	0.027	0.210	0.002	0.016										
SNa	0.22	0.12	0.53 *	0.35	0.63 **	0.63 **									
*p*-value	0.360	0.615	0.016	0.135	0.003	0.003									
RNa	0.49 *	0.56 *	0.34	0.47 **	0.59 **	0.89 **	0.51 *								
*p*-value	0.025	0.010	0.142	0.034	0.005	0.000	0.021								
SMg	0.51 *	0.37	0.90 **	0.13	0.95 **	0.40	0.62 **	0.45 *							
*p*-value	0.019	0.108	0.000	0.600	0.000	0.079	0.003	0.045							
RMg	0.09	0.14	0.11	0.92 **	0.18	0.73 **	0.31	0.61 **	0.14						
*p*-value	0.712	0.554	0.651	0.000	0.442	0.000	0.178	0.004	0.564						
SMn	0.52 *	0.38	0.86 **	0.22	0.88 **	0.43	0.50 *	0.43	0.91 **	0.22					
*p*-value	0.018	0.098	0.000	0.363	0.000	0.053	0.024	0.056	0.000	0.359					
RMn	0.35	0.32	0.21	0.54 *	0.27	0.66 **	0.48 *	0.52 *	0.27	0.65 **	0.36				
*p*-value	0.130	0.171	0.372	0.012	0.242	0.001	0.031	0.017	0.245	0.002	0.122				
SCu	−0.20	−0.09	0.41	0.06	0.19	0.02	0.33	0.09	0.25	−0.03	0.11	0.20			
*p*-value	0.388	0.712	0.074	0.806	0.429	0.939	0.160	0.694	0.280	0.918	0.660	0.402			
RCu	−0.10	−0.05	0.00	0.83 **	−0.04	0.42	0.28	0.16	0.02	0.78 **	0.10	0.61 **	0.05		
*p*-value	0.669	0.831	0.992	0.000	0.872	0.067	0.234	0.501	0.926	0.000	0.671	0.004	0.835		
SB	0.46 *	0.21	0.48 *	−0.16	0.64 **	0.23	0.36	0.36	0.56 *	−0.06	0.49 *	−0.17	−0.13	−0.27	
*p*-value	0.041	0.373	0.031	0.503	0.002	0.337	0.121	0.125	0.010	0.808	0.028	0.469	0.594	0.245	
RB	0.45 *	0.37	0.17	0.23	0.51 *	0.62 **	0.31	0.71 **	0.37	0.42	0.31	0.11	−0.28	0.03	0.58 **
*p*-value	0.043	0.109	0.462	0.337	0.021	0.003	0.181	0.000	0.109	0.065	0.189	0.633	0.232	0.910	0.007

## Data Availability

Not applicable.

## Supplementary Materials

The following supporting information can be downloaded at: https://www.mdpi.com/article/10.3390/biology11081094/s1. Table S1: Means (in mg plant^−1^) of the RP (root phosphorus), SP (shoot phosphorus), RNa (root sodium), and SNa (shoot sodium) uptake of 19 Aegilops genotypes and Bolal 2973, the B tolerant check genotype in Control (3.1 μM B), 10 ppm B (toxic B), and 100 ppm B (highly toxic B), Table S2: Means (in mg plant^−1^) of the RMg (root magnesium), and SMg (shoot magnesium) uptake, and the means (in µg plant^−1^) of RMn (root manganese), and SMn (shoot manganese) uptake of 19 Aegilops accessions and Bolal 2973, the B tolerant check genotype in Control (3.1 μM B), 10 ppm B (toxic B), and 100 ppm B (highly toxic B), Table S3: Means (in µg plant^−1^) of the RCu (root copper), SCu (shoot copper), RB (root boron), and SB (shoot boron) uptake of 19 Aegilops accessions and Bolal 2973, the B tolerant check genotype in Control (3.1 μM B), 10 ppm B (toxic B), and 100 ppm B (highly toxic B). Table S4: Means of the RDW (root dry weight), SDW (shoot dry weight), and the means (in mg plant^−1^) of RCa (root calcium) uptake, and SCa (shoot calcium) uptake of 19 Aegilops accessions and Bolal 2973, the B tolerant check genotype in Control (3.1 μM B), 10 ppm B (toxic B), and 100 ppm B (highly toxic B).
