# Supplementary material for: Nutrient Homeostasis of Aegilops Accessions Differing in B Tolerance Level under Boron Toxic Growth Conditions
Source: Biology (Basel). 2022 Jul 22;11(8):1094. doi: 10.3390/biology11081094 (PMC9331359; doi:10.3390/biology11081094)
Supplement: Supplementary file 1 [file biology-11-01094-s001.zip › biology-1783570-supplementary.pdf]

Supplementary Material

Supplementary Table S1: Means (in mg plant^{-1}) of the RP (root phosphorus), SP (shoot phosphorus), RNa (root sodium), and SNa (shoot sodium) uptake of 19 Aegilops accessions and the B tolerant check cultivar, Bolal 2973 grown under Control (3.1 µM B), toxic B (10 ppm B) and highly toxic B (100 ppm B) .

Code	Shoot Phosphorus			Root Phosphorus			Shoot Sodium			Root Sodium		
	Control	10 ppm	100 ppm	Control	10 ppm	100 ppm	Control	10 ppm	100 ppm	Control	10 ppm	100 ppm
**Ab1**	0.229	0.177	0.179	0.108	0.094	0.155	0.017	0.046	0.030	0.021	0.020	0.042
**Ab2**	0.255	0.298	0.267	0.000	0.018	0.036	0.013	0.035	0.030	0.007	0.005	0.016
**Ab3**	0.233	0.173	0.199	0.076	0.123	0.147	0.016	0.014	0.022	0.009	0.016	0.042
**Ac1**	0.535	0.826	0.301	0.147	0.149	0.037	0.017	0.032	0.037	0.060	0.040	0.019
**Ac2**	0.299	0.361	0.293	0.099	0.031	0.024	0.016	0.030	0.017	0.014	0.010	0.011
**Ac3**	0.325	0.259	0.200	0.103	0.051	0.025	0.029	0.019	0.038	0.059	0.027	0.019
**Ac4**	0.362	0.276	0.718	0.168	0.103	0.083	0.017	0.018	0.040	0.019	0.023	0.045
**Ac5**	0.229	0.225	0.127	0.082	0.056	0.028	0.011	0.014	0.015	0.039	0.019	0.023
**As1**	0.121	0.077	0.093	0.058	0.021	0.017	0.012	0.011	0.029	0.046	0.027	0.018
**As2**	0.236	0.126	0.088	0.084	0.036	0.015	0.036	0.011	0.025	0.053	0.019	0.016
**Al1**	0.116	0.125	0.117	0.023	0.036	0.020	0.011	0.012	0.019	0.012	0.026	0.014
**Al2**	0.257	0.285	0.117	0.072	0.077	0.022	0.015	0.024	0.019	0.046	0.061	0.020
**At1**	0.238	0.193	0.154	0.028	0.041	0.034	0.010	0.010	0.020	0.008	0.007	0.016
**At2**	0.138	0.241	0.181	0.043	0.078	0.043	0.009	0.020	0.022	0.009	0.031	0.020
**At3**	0.390	0.202	0.139	0.065	0.043	0.030	0.024	0.016	0.038	0.034	0.014	0.028
**At4**	0.487	0.537	0.250	0.128	0.101	0.054	0.018	0.025	0.024	0.025	0.016	0.021
**Au1**	0.184	0.226	0.179	0.044	0.066	0.034	0.014	0.017	0.027	0.020	0.021	0.029
**Au2**	0.174	0.315	0.267	0.083	0.123	0.048	0.007	0.015	0.031	0.018	0.021	0.020
**Au3**	0.148	0.196	0.141	0.073	0.095	0.037	0.011	0.019	0.036	0.025	0.031	0.040
**Bolal**	0.602	0.581	0.593	0.132	0.126	0.075	0.037	0.038	0.075	0.048	0.038	0.044

Supplementary Table S2: Means (in mg plant^{-1}) of the RMg (root magnesium) and SMg (shoot magnesium) uptake, and the means (in µg plant^{-1}) of RMn (root manganese), and SMn (shoot manganese) uptake of 19 Aegilops accessions and the B tolerant check cultivar, Bolal 2973 grown under Control (3.1 µM B), toxic B (10 ppm B) and highly toxic B (100 ppm B).

Code	Shoot Magnesium			Root Magnesium			Shoot Manganese			Root Manganese		
	Control	10 ppm	100 ppm	Control	10 ppm	100 ppm	Control	10 ppm	100 ppm	Control	10 ppm	100 ppm
Ab1	0.083	0.079	0.069	0.025	0.023	0.024	0.99	0.97	0.94	0.51	0.58	0.42
Ab2	0.094	0.100	0.084	0.018	0.005	0.009	1.20	1.43	1.18	0.31	0.11	0.15
Ab3	0.097	0.074	0.074	0.025	0.032	0.036	1.13	0.89	0.92	0.41	0.59	0.61
Ac1	0.105	0.171	0.070	0.063	0.066	0.013	5.21	6.59	2.57	1.02	1.13	0.62
Ac2	0.090	0.114	0.084	0.025	0.009	0.005	1.96	2.10	1.27	0.38	0.17	0.08
Ac3	0.078	0.063	0.063	0.025	0.022	0.014	2.75	2.37	2.20	2.08	0.98	0.81
Ac4	0.123	0.081	0.166	0.054	0.042	0.027	2.46	2.07	4.10	1.02	0.81	0.44
Ac5	0.062	0.071	0.042	0.034	0.024	0.012	3.53	3.72	2.02	0.76	0.57	0.81
As1	0.042	0.028	0.042	0.024	0.013	0.009	1.58	0.91	1.07	1.60	0.67	0.40
As2	0.062	0.027	0.029	0.033	0.018	0.010	2.67	1.05	0.85	1.31	0.96	0.38
Al1	0.036	0.033	0.035	0.009	0.014	0.007	1.45	1.33	1.07	0.54	1.50	0.59
Al2	0.071	0.082	0.037	0.022	0.027	0.008	3.41	3.60	1.65	2.24	3.23	0.89
At1	0.050	0.038	0.038	0.006	0.010	0.011	1.93	1.80	1.41	0.46	0.72	0.78
At2	0.039	0.069	0.042	0.011	0.027	0.013	1.93	3.30	1.78	0.76	1.78	1.08
At3	0.099	0.044	0.040	0.026	0.019	0.017	4.41	1.84	0.98	1.04	0.72	0.39
At4	0.086	0.094	0.057	0.040	0.022	0.027	2.86	3.23	1.57	0.88	0.62	0.80
Au1	0.050	0.056	0.045	0.017	0.022	0.011	1.81	2.49	1.55	0.35	0.30	0.32
Au2	0.047	0.078	0.059	0.029	0.036	0.014	1.99	3.54	1.77	0.59	0.75	0.54
Au3	0.055	0.078	0.062	0.029	0.054	0.020	2.19	2.18	2.11	0.43	0.68	0.88
Bolal	0.134	0.124	0.177	0.034	0.032	0.018	4.49	4.27	4.71	2.73	2.04	3.64

Supplementary Table S3: Means (in µg plant^{-1}) of the RCu (root copper), SCu (shoot copper), RB (root boron), and SB (shoot boron uptake of 19 Aegilops accessions and the B tolerant check cultivar, Bolal 2973 grown under Control (3.1 µM B), toxic B (10 ppm B) and highly toxic B (100 ppm B).

Code	Shoot Copper			Root Copper			Shoot Boron			Root Boron		
	Control	10 ppm	100 ppm	Control	10 ppm	100 ppm	Control	10 ppm	100 ppm	Control	10 ppm	100 ppm
**Ab1**	2.60	4.34	0.98	5.21	4.44	5.34	0.61	16.59	83.01	0.33	1.52	16.97
**Ab2**	2.66	2.56	1.18	2.65	0.69	1.19	0.51	18.51	131.24	0.07	0.21	3.78
**Ab3**	2.41	1.43	0.82	3.35	4.48	5.26	0.53	15.93	105.71	0.11	1.87	17.80
**Ac1**	1.52	1.98	0.84	3.49	3.40	1.28	0.81	35.02	101.48	0.32	2.03	3.26
**Ac2**	1.60	2.00	0.90	3.84	1.14	0.65	0.55	13.15	130.97	0.06	0.64	2.36
**Ac3**	3.19	0.87	0.97	1.13	1.49	1.24	0.55	10.64	66.36	0.10	0.39	1.62
**Ac4**	2.75	1.30	1.64	7.82	5.84	4.07	0.56	16.27	295.62	0.14	2.24	15.46
**Ac5**	0.77	0.76	0.69	1.97	1.17	1.44	0.71	18.54	52.25	0.14	0.60	1.63
**As1**	0.69	0.59	0.90	0.72	0.95	1.05	0.28	4.88	49.72	0.08	0.16	1.38
**As2**	1.68	0.48	1.11	2.18	1.25	1.15	0.30	4.97	30.52	0.15	0.55	1.47
**Al1**	0.67	0.62	0.57	0.56	1.54	1.01	0.45	4.56	42.65	0.03	0.27	0.75
**Al2**	0.99	1.27	0.82	2.59	2.27	1.20	0.60	13.73	55.60	0.13	0.67	0.98
**At1**	0.61	0.59	0.87	0.56	0.82	0.91	0.40	7.84	43.57	0.05	0.25	1.95
**At2**	0.51	0.96	0.74	1.37	2.50	1.16	0.51	7.78	52.52	0.08	0.50	1.46
**At3**	1.47	0.92	0.71	1.70	1.16	1.18	0.77	8.52	72.95	0.22	0.48	5.91
**At4**	1.21	1.38	0.75	1.24	0.57	0.95	0.63	23.45	73.71	0.13	1.30	3.31
**Au1**	0.88	1.18	0.63	1.14	1.50	1.25	0.33	9.89	74.23	0.13	0.48	3.34
**Au2**	0.53	1.14	0.83	1.45	1.52	0.63	0.30	19.35	106.75	0.08	0.92	3.97
**Au3**	0.71	0.94	1.00	1.26	1.46	1.63	0.46	19.95	84.30	0.19	1.49	2.59
**Bolal**	0.79	0.71	0.94	1.24	0.86	0.89	1.18	17.31	129.68	0.52	1.04	6.04

Supplementary Table S4: Means of the RDW (root dry weight), SDW (shoot dry weight), and the means (in mg plant^{-1}) of RCa (root calcium) uptake, and SCa (shoot calcium) uptake of 19 Aegilops accessions and the B tolerant check cultivar, Bolal 2973 grown under Control (3.1 µM B), toxic B (10 ppm B) and highly toxic B (100 ppm B).

Code	Shoot Dry Weight			Root Dry Weight			Shoot Calcium			Root Calcium		
	Control	10 ppm	100 ppm	Control	10 ppm	100 ppm	Control	10 ppm	100 ppm	Control	10 ppm	100 ppm
**Ab1**	0.030	0.033	0.032	0.009	0.008	0.007	0.153	0.167	0.128	0.050	0.049	0.062
**Ab2**	0.027	0.035	0.039	0.009	0.011	0.008	0.184	0.219	0.163	0.028	0.010	0.021
**Ab3**	0.034	0.030	0.033	0.010	0.009	0.007	0.180	0.132	0.130	0.049	0.048	0.065
**Ac1**	0.043	0.046	0.040	0.015	0.014	0.008	0.200	0.278	0.141	0.169	0.152	0.048
**Ac2**	0.040	0.043	0.033	0.012	0.012	0.007	0.150	0.196	0.135	0.043	0.022	0.011
**Ac3**	0.026	0.024	0.018	0.006	0.006	0.002	0.198	0.145	0.154	0.088	0.068	0.080
**Ac4**	0.029	0.036	0.036	0.009	0.009	0.008	0.190	0.150	0.305	0.065	0.050	0.055
**Ac5**	0.031	0.033	0.024	0.011	0.010	0.006	0.135	0.140	0.086	0.093	0.062	0.053
**As1**	0.018	0.018	0.010	0.007	0.005	0.002	0.123	0.092	0.129	0.071	0.065	0.055
**As2**	0.018	0.018	0.014	0.006	0.005	0.003	0.233	0.083	0.138	0.218	0.064	0.095
**Al1**	0.012	0.013	0.010	0.003	0.003	0.002	0.099	0.088	0.083	0.019	0.078	0.043
**Al2**	0.029	0.028	0.015	0.009	0.007	0.003	0.172	0.203	0.119	0.071	0.082	0.068
**At1**	0.030	0.021	0.020	0.008	0.005	0.004	0.109	0.085	0.116	0.031	0.034	0.077
**At2**	0.022	0.023	0.021	0.006	0.006	0.005	0.077	0.142	0.103	0.030	0.072	0.047
**At3**	0.034	0.019	0.020	0.008	0.004	0.003	0.249	0.120	0.101	0.117	0.080	0.106
**At4**	0.041	0.042	0.032	0.012	0.011	0.004	0.200	0.221	0.117	0.074	0.059	0.043
**Au1**	0.026	0.022	0.024	0.008	0.007	0.005	0.132	0.164	0.102	0.077	0.100	0.060
**Au2**	0.033	0.038	0.028	0.015	0.015	0.006	0.103	0.187	0.135	0.074	0.063	0.040
**Au3**	0.029	0.034	0.025	0.012	0.015	0.006	0.130	0.156	0.158	0.085	0.120	0.105
**Bolal**	0.053	0.042	0.053	0.011	0.009	0.007	0.221	0.223	0.353	0.119	0.103	0.088
